# An Electrochemical Gas Biosensor Based on Enzymes Immobilized on Chromatography Paper for Ethanol Vapor Detection

**DOI:** 10.3390/s17020281

**Published:** 2017-02-01

**Authors:** Tatsumi Kuretake, Shogo Kawahara, Masanobu Motooka, Shigeyasu Uno

**Affiliations:** Department of Electrical and Electronic Engineering, Ritsumeikan University, Kusatsu, Shiga 525-8577, Japan; t.kuretake@gmail.com (T.K.); re0039ke@ed.ritsumei.ac.jp (S.K.); re0064vp@ed.ritsumei.ac.jp (M.M.)

**Keywords:** electrochemical sensor, chromatography paper-based sensor, gas sensor

## Abstract

This paper presents a novel method of fabricating an enzymatic biosensor for breath analysis using chromatography paper as enzyme supporting layer and a liquid phase layer on top of screen printed carbon electrodes. We evaluated the performance with ethanol vapor being one of the breathing ingredients. The experimental results show that our sensor is able to measure the concentration of ethanol vapor within the range of 50 to 500 ppm. These results suggest the ability of detecting breath ethanol, and it can possibly be applied as a generic vapor biosensor to a wide range of diseases.

## 1. Introduction

Nowadays, breath analysis for disease diagnosis has attracted attention because it represents a noninvasive diagnostics methodology. Furthermore, it was proven that human breath contains biomarkers that can be used to diagnose diseases such as diabetes, lung cancer and halitosis [1]. Thus, it is now possible to monitor one’s body condition simply through breathing [2,3,4].

Many types of gas sensors such as the semiconductor type have been investigated and developed so far [5,6,7]. The advantages of this type of sensor are its sensitivity and good response towards target molecules in the gas phase. This type of sensor responds to the target substances by the physical adsorption on the surface of semiconductor material, resulting in a change in the electric resistance [8]. This principle, however, does not provide good specificity, leading to a false result. This issue has been addressed in the previous reports by targeting the substances using their specific enzymes [9,10,11]. These sensors measure electrical signals such as the current output due to the chemical reactions between the enzymes and target molecules. Such enzyme electrodes are fabricated by immobilizing a thin layer of enzyme at the electrode surface. Hence, the immobilization method used plays an important role in the fabrication of enzyme electrodes. Various methods have been reported [12,13,14,15,16,17] and generally cross-linking and entrapment method are often used [9,11,15,16,17].

However, these fabrication methods are time-consuming because of the complicated process required for immobilizing the enzyme layer, such as irradiating the thin layer of enzyme with a fluorescent light [15,16,17]. In order to complete the process, there are many procedures that need to be done. Additionally, enzyme reactions do not occur under dry conditions, therefore, membranes such as filter paper or a gel matrix must be attached to the electrode surface after placing the enzyme layer [11]. This layer must be wet so as to prevent the performance degradation of the enzyme electrode. Thus, previous methods for fabricating enzyme electrode gas biosensors have had problems of requiring considerable time in preparing the enzyme layer on the electrodes.

We propose to use chromatography paper (ChrPr) as an enzyme and water supporting layer in an enzyme electrode for gas detection. Since chromatography paper is a porous material consisting of cellulose fiber, it can be used as an enzyme supporting layer by simply soaking it in an enzyme solution and then drying it. The dried enzyme supporting layer will be wetted prior to measurement. As chromatography paper has high water absorbency properties, wet conditions can easily be realized by just putting it in contact with the other solution. Our new method using ChrPr therefore provides a simple but effective method to fabricate an enzyme electrode for gaseous detection.

In this study, we propose a new type of enzyme electrode for detecting substances in the gaseous phase based on ChrPr enzyme immobilized on screen printed carbon electrodes. With ethanol vapor being one of the ingredients of breath, we demonstrate the response, sensitivity, correlation of output current with concentration, and its reproducibility. We also performed experiments to evaluate the feasibility of modified ChrPr enzyme electrodes (ChrSPCEs).

## 2. Materials and Methods 

**Figure 1:**
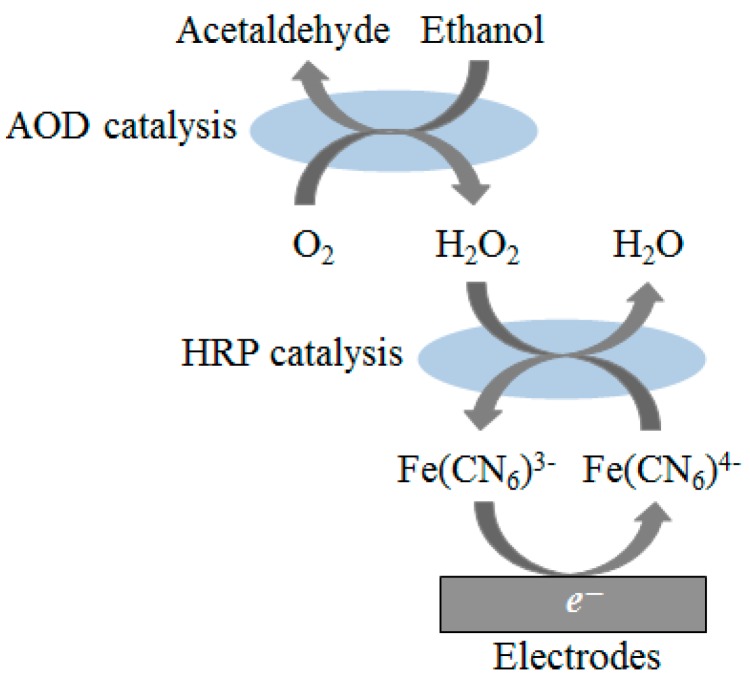
Enzymatic reaction at the surface of ChrSPCEs in ethanol detection.

We adopted the ethanol reaction with two enzymes, alcohol oxidase (AOD) and peroxidase (HRP), and an electron mediator [Fe(CN)_6_]^4−^ (Ferro) [18], as shown Figure 1. Alcohol oxidase oxidizes ethanol to acetaldehyde using oxygen (O_2_) as the electron acceptor, producing hydrogen peroxide as byproduct, as shown in the following reaction:

CH_3_CH_2_OH + O_2_ → CH_3_CHO + H_2_O_2_ (1)

Subsequently, the HRP reduces hydrogen peroxide to H_2_O using Ferro as electron donor, producing [Fe(CN)_6_]^3−^ (Ferri) as shown below:

H_2_O_2_ + 2[Fe(CN)_6_]^4−^ + 2H^+^ → 2H_2_O + 2[Fe(CN)_6_]^3−^ (2)

The reduction current of Ferri is then measured at the working electrode (WE) as follows:

[Fe(CN)_6_]^3−^ + e^−^ → [Fe(CN)_6_]^4−^ (3)

Thus, the reduction current of Ferri becomes proportional to ethanol concentration. The enzyme supporting layer is constructed with two different layers of paper, namely, an enzyme layer and a mediator layer. When both mediator and enzyme are immobilized in the same layer, the mediator can be oxidized by natural peroxide substances, generating electro-active substances which can be observed as background current. By separating the enzyme and mediator layers, this background current can be reduced.

**Figure 2:**
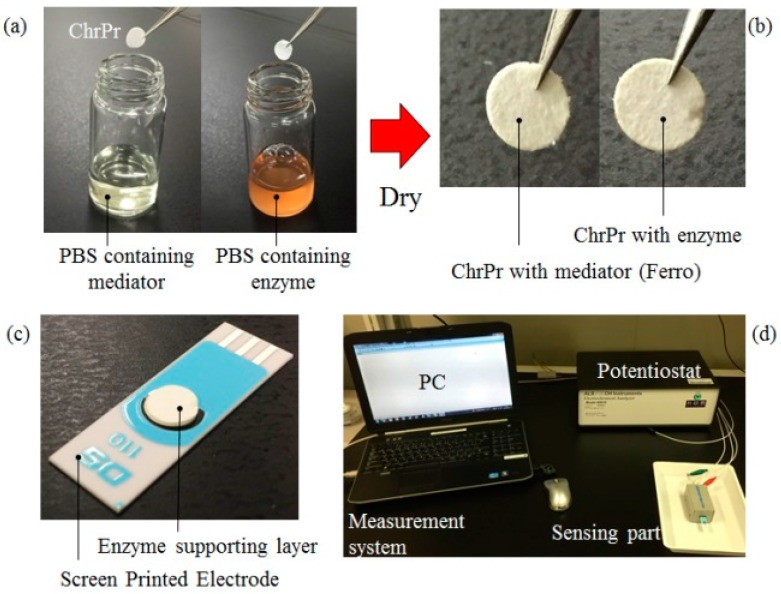
Enzyme supporting layer is a lamination of enzyme layer and mediator layer. (**a**) ChrPrs dipped into the solutions containing AOD and HRP or Ferro; (**b**) ChrPrs left to dry in a refrigerator at 4 °C for 12 h, respectively; (**c**) Modified ChrPr enzyme electrodes (ChrSPCEs) placed onto the screen-printed electrodes; (**d**) Measurement setup of ethanol gaseous analysis by the ChrSPCEs.

In this study, chromatography paper (ChrPr: 1Chr, Catalog No. 3001-861, Whatman, GE Healthcare, Tokyo, Japan) was used as the enzyme supporting layer. Firstly, ChrPr was cut in a circle of 6 mm diameter. For the enzyme layer, ChrPrs were modified by dipping them into solution containing phosphate buffer solution, prepared by mixing K_2_HPO_4_ and KH_2_PO_4_ (PBS: 100 mM, pH = 7.0, Wako, Osaka, Japan), alcohol oxidase (AOD: 70 U/mL, From *Pichia pastoris*, E.C. 1.1.3.13, Sigma Aldrich Japan, Tokyo, Japan) and peroxidase (HRP: 70 U/mL, From horseradish, E.C. 1.11.1.7, Wako). For mediator layer, ChrPrs were modified by just dipping them into a solution containing PBS and reducing mediator (Ferro: K_4_[Fe(CN)_6_], 100 mM, Wako). After they were left to dry in the refrigerator at 4 °C for 12 h, the AOD, HRP and Ferro were immobilized in ChrPr, as shown in Figure 2a,b. Modified ChrPr enzyme electrodes (ChrSPCEs) were constructed by placing modified ChrPr fabricated in the way mentioned above on a screen printed carbon electrodes (SPCEs: DRP-110, Dropsens, Asturias, Spain), as shown in Figure 2c. The sample vapor with ethanol concentrations at 0, 50, 100, 200, 500 ppm were used in measurement. We prepared the vapor with ethanol (WEth: 50—500 ppm) by volatilizing ethanol solutions (C_2_H_6_O, 99.5 % v/v, Wako) in a sampling bag (F2s (PVDF), 1-6332-12, ASONE, Osaka, Japan) filled with air without ethanol (WoEth: 0 ppm). Figure 2d shows the measurement setup of the ethanol vapor analysis. A chronoamperometry (CA) method was performed by applying −200 mV step potential vs. Ag/AgCl to the working electrode. The 12 μL of PBS without enzymes or ethanol was dropped on the dried enzyme supporting layer, and step potential was applied to measure electric current. The 20 mL of sample vapor was then blown onto the wet enzyme supporting layer for 20 s. A computer controlled potentiostat (ALS/CH Electrochemical Analyzer Model 610DR, BAS, Tokyo, japan) was used to evaluate the reduction current of Ferri produced by the AOD and HRP enzymatic reaction.

## 3. Results and Discussion

**Figure 3:**
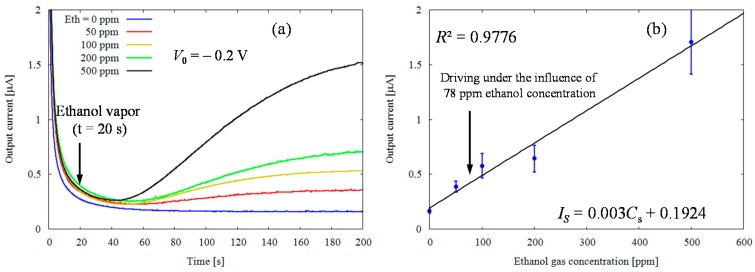
Typical current responses of modified chromatography paper enzyme electrodes for several ethanol gaseous concentrations. (**a**) The chronoamperometory at V_0_ = −0.2 V with 0, 50, 100, 200 and 500 ppm; (**b**) The calibration curve of the reduction current taken at t = 200 s by enzymatic catalyst on the concentration of ethanol gas.

Figure 3a shows CA measurement results. No significant changes were observed when vapor without ethanol (Eth = 0 ppm) is blown onto the ChrSPCEs as opposed to the case when the vapor with ethanol is blown, where an obvious increase in reduction current can be seen. After vapor with ethanol was released, the output current started to increase linearly for 80 s and then observed to reach a steady state at t = 200 s for all concentrations of ethanol vapor. This indicates the reducing current produced by the oxidation of ethanol molecules diffused from the sample vapor into PBS. A time of about 40 s was required for the output current caused by the enzyme reactions to increase.

Figure 3b indicates the calibration curve result. From the graph, the output current taken at t = 200 s was positively correlated with ethanol vapor concentration up to 0 to 500 ppm (coefficient of determination = 0.9776, n = 5). The regression equation of calibration curve was deduced by least square method as shown below:

*I*_S_ = 0.003 Cs + 0.1924, (4)

where I_S_ is the output current (μA), and Cs is the initial concentration of gaseous ethanol in ppm. The calibration range covers the alcohol levels of driving under the influence (78 ppm) [9,19,20]. These results suggest that our sensor can potentially be used to measure concentration of ethanol in expiration with feasible sensitivity.

The reproducibility of the sensor in ethanol vapor (50 ppm) can be evaluated by coefficients of variation (CV). The CV value of the sensor was 12.6% (n = 5), demonstrating that our sensor has a reasonably good reproducibility.

It is important to note that our sensor can be used for detection of various types of targets by just changing the type of enzyme used. Gas sensors for the diagnosis of diseases such as cancer, sick-house syndrome or diabetes are reported [1,2]. Some specific examples exploiting enzymatic reactions are formaldehyde [21], acetone [22], and lactate [23]. These might be helpful for noninvasive and safe diagnosis of diseases in the future.

## 4. Conclusions 

A new type of enzyme electrodes, ChrSPCEs which are based on a modified chromatography paper-immobilized enzyme has been designed. It is being evaluated by using the ethanol vapor in order to certify its performance as a potential biosensor for gaseous analysis. A 78 ppm concentration of ethanol vapor is identified as driving under the influence of alcohol. As ChrSPCEs can detect ethanol vapor ranging in concentrations from 50 to 500 ppm, the results obtained in this paper, suggest that ChrSPCEs is able to measure the ethanol in exhaled air. Finally, we discussed the significance of our results and how this study might be useful in the near future. The ChrSPCEs can be applied as vapor biosensors to a wide range of diseases by altering the type of enzyme immobilized in ChrPr.

## Supplementary Materials

The following are available online at www.mdpi.com/1424-8220/17/2/281/s1, Figure S1: Sensor performance degradation during storage, Figure S2: Cyclic voltammograms, Figure S3: Enzymatic current in cyclic voltammograms.
