# Supplementary material for: An Electrochemical Gas Biosensor Based on Enzymes Immobilized on Chromatography Paper for Ethanol Vapor Detection
Source: Sensors (Basel). 2017 Feb 1;17(2):281. doi: 10.3390/s17020281 (PMC5336011; doi:10.3390/s17020281)
Supplement: Supplementary file 1 [file sensors-17-00281-s001.pdf]

Supplementary Materials: An electrochemical Gas Biosensor based on Enzymes Immobilized on Chromatography Paper for Ethanol Vapor Detection

Tatsumi Kuretake, Shogo Kawahara, Masanobu Motooka, and Shigeyasu Uno

Figure S1 shows the output currents at t = 200s in the chronoamperometory measurements as described in Figure 3. The chromatography paper sensors were fabricated on day 1, stored in a container with silica gel as desiccant, and used in the measurements repeated on the following three days. This experimental data indicates sensor stability, or shelf lifetime. Apart from the variation due to hand technique, the sensor output appears to be gradually decreasing. This might be due to degradation of enzymes (AOD, HRP), as $[Fe(CN)_6]^{4-}$ has quite long shelf time when it is dry and kept in dark. Implications from these results are as follows: (a) For laboratory use, experiments should be done on the same day of sensor fabrication, (b) for use as a commercial product, some preserving technique must be developed.

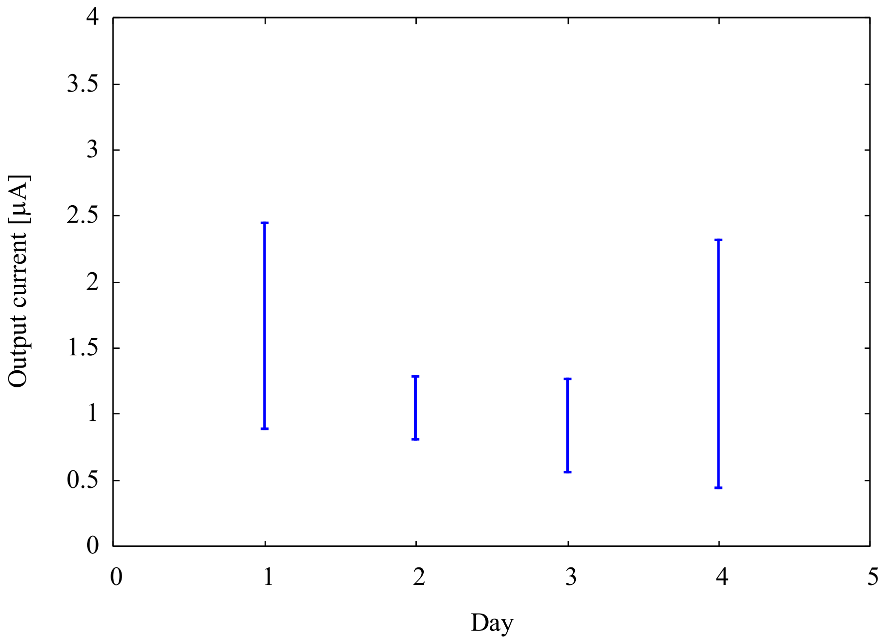

Figure S1. Output current at t = 200 s in the chronoamperometory (CA) measurement with PBS and ethanol gas of 500 ppm, repeated for four consecutive days with the sensors fabricated on day 1. In each day, five CA measurements were repeated to calculate the average and standard deviations.

Figure S2 shows the cyclic voltammogram (CV) obtained by using the chromatography paper enzyme electrode.

First, only mediator layer was placed on the SPCE, and CV was obtained by dropping PBS 6μL on the mediator layer, which gave the navy curve in the figure. The enzyme layer is omitted in this measurement to isolate the current due only to the electron transfer between $[Fe(CN)_6]^{4-}/[Fe(CN)_6]^{3-}$ molecules and carbon electrode. The potential sweep starts at −0.4 V, continues up to +0.7 V, and comes back to −0.4 V. The curve shows the oxidation current peak at +0.650 V and reduction current peak at −0.120 V, which give the half-wave potential $E_{1/2}$ = +0.385 V. It can be approximately considered as the redox potential of $[Fe(CN)_6]^{4-}/[Fe(CN)_6]^{3-}$ in this electrochemical system.

Next, measurement was carried out by dropping PBS 12 μL on the mediator layer and enzyme layer placed on top of the SPCE, which gave the blue curve in the figure. The oxidation and reduction current peaks appeared at +0.607 V and −0.088 V, respectively, which indicates

$E_{1/2}$ = +0.348 V. Note that the redox potential is almost unchanged, and there are no additional current peaks observed. This indicates that the electron transfer to/from the electrode is fully dominated by that of $[Fe(CN)_6]^{4-}/[Fe(CN)_6]^{3-}$, not by the direct electron transfer with enzymes.

Finally, a new set of mediator/enzyme layers was placed on the carbon electrode, PBS was dropped on the paper, and then the ethanol vapor gas of 500 ppm was injected on it. The cyclic voltammogram was taken 180 s after the gas injection, and the result is shown by the red curve in the figure. Note that the voltammogram again shows the oxidation and reduction current peaks at similar potentials as the one without ethanol gas, and there are no additional current peaks.

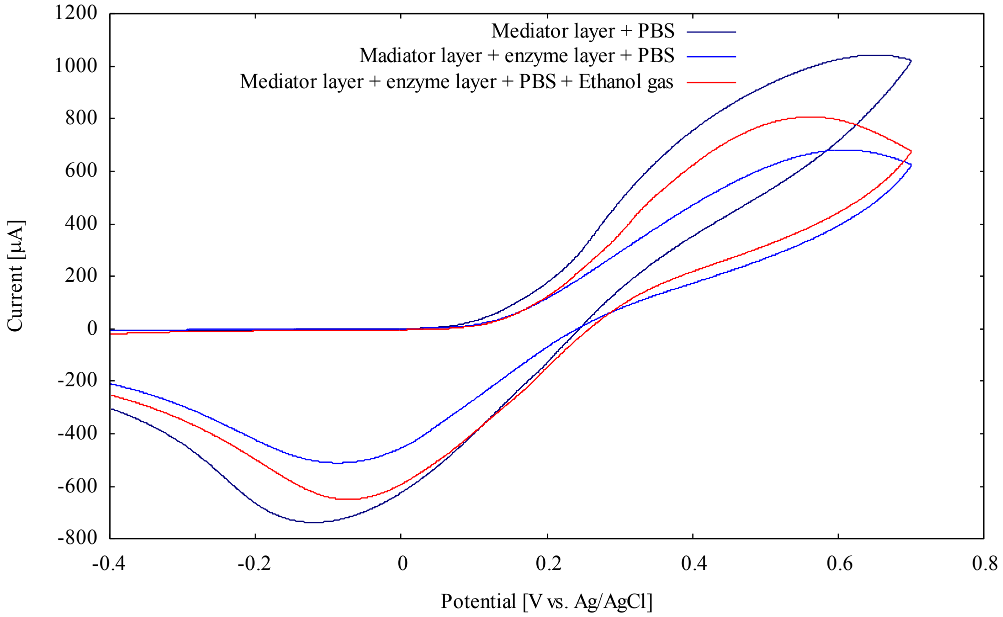

Figure S2. Cyclic voltammogram obtained by only mediator layer + PBS (navy), mediator layer + enzyme layer + PBS (blue), and mediator layer + enzyme layer + PBS + ethanol gas (red). Potential sweep rate is 0.1 V/s, and other reagent/fabrication conditions are the same as the paper.

Figure S3 shows the same CV data as Figure S2, but zoomed up in the potential sweep segment from −0.4 V to 0.1 V. Note that the current shown here reflects $[Fe(CN)_6]^{3-}$ content in the solution, and especially the current shown by the red curve mainly comes from the $[Fe(CN)_6]^{3-}$ molecules produced by the ethanol-enzyme reaction described by Equations (1)–(3) in the paper.

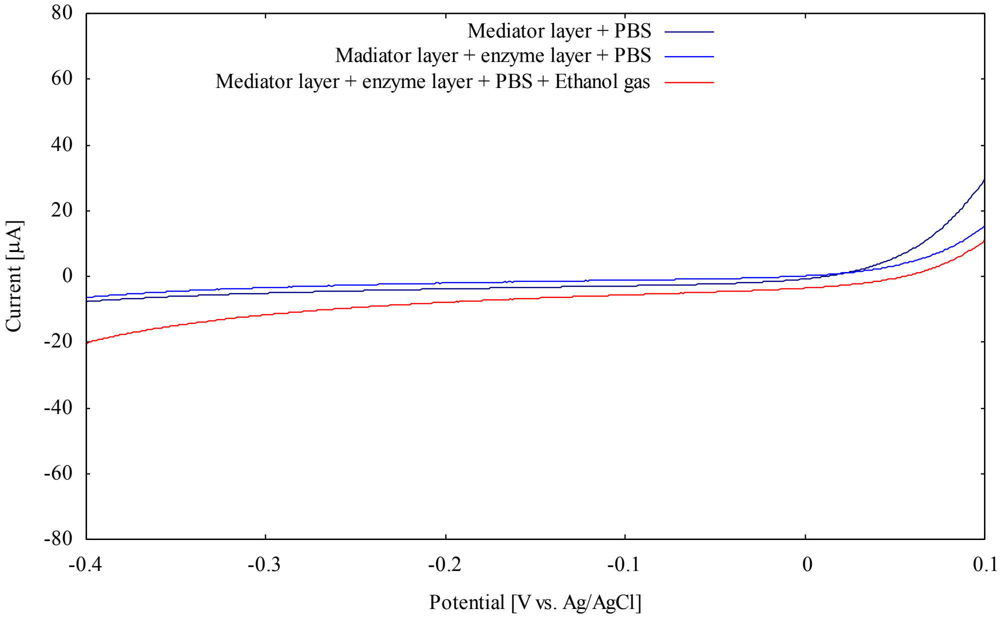

Figure S3. Cyclic voltammogram obtained by only mediator layer + PBS (navy), mediator layer + enzyme layer + PBS (blue), and mediator layer + enzyme layer + PBS + ethanol gas (red). Potential sweep rate is 0.1 V/s, and other reagent/fabrication conditions are the same as the paper.

Based on the results shown in Figures S2 and S3, the choice of the constant potential $V_0 = -200$ mV in the chronoamperometory measurements is valid, because it is well below the redox potential of $[Fe(CN)_6]^{4-}/[Fe(CN)_6]^{3-}$, as well as its reduction current peak potential.
